# Good and bad sides of TGFβ-signaling in myocardial infarction

**DOI:** 10.3389/fphys.2015.00066

**Published:** 2015-03-04

**Authors:** Gerhild Euler

**Affiliations:** Institute of Physiology, Justus-Liebig-UniversityGiessen, Germany

**Keywords:** myocardial infarction, transforming growth factor beta, SMAD, microRNA, hypertrophy, apoptosis, fibrosis, TAK1

## Abstract

Myocardial infarction is a prevailing cause of death in industrial countries. In spite of the good opportunities we have nowadays in interventional cardiology to reopen the clotted coronary arteries for reperfusion of ischemic areas, post-infarct remodeling emerges and contributes to unfavorable structural conversion processes in the myocardium, finally resulting in heart failure. The growth factor TGFβ is upregulated during these processes. In this review, an overview on the functional role of TGFβ signaling in the process of cardiac remodeling is given, as it can influence apoptosis, fibrosis and hypertrophy thereby predominantly aggravating ischemia/reperfusion injury.

## Introduction

Myocardial infarction is one of the most life-threatening diseases in industrial countries. Severe ischemia causes immediate necrotic cell death. Nevertheless timely reperfusion of ischemic areas also causes cell damage and promotes post-infarct remodeling finally leading to heart failure.

Post-infarct remodeling is a multifaceted structural conversion process in the myocardium comprising loss of cardiomyocytes by programmed cell death that is accompanied by compensatory induction of hypertrophic growth and fibrosis. All together these events result in adverse myocardial structures with progressive dilatation and reduced pump function.

Interestingly, the cytokine transforming growth factor beta (TGFβ) has been described to influence each of the single components of the remodeling process, i.e., TGFβ1 promotes myocardial fibrosis (Okada et al., 2005; Edgley et al., 2012), cardiomyocyte apoptosis (Schneiders et al., 2005) or cardiac hypertrophy (Huntgeburth et al., 2011). In addition, TGFβ up-regulation after myocardial infarction has been described by several groups (Hao et al., 1999; Vilahur et al., 2011; Li et al., 2012), and a significant relationship between reduced ejection fractions in patients after acute myocardial infarction and increases in TGFβ levels was found (Talasaz et al., 2013).

All these correlative findings between myocardial infarction and induction of TGFβ suggest contribution of TGFβ to post-infarct remodeling. In this review we now have a detailed look on the role of TGFβ during and after myocardial infarction.

## TGFβ signaling pathways

Of the three different TGFβ isoforms (1, 2, and 3) that have been identified, TGFβ1 is the most prominent and most often analyzed form. In the heart several cell types are identified as source of TGFβ release, as cardiomyocytes, endothelial cells, fibroblasts and macrophages can release TGFβ. Free oxygen radicals, as they are found in myocardial infarction, can induce TGFβ activation. Talasaz and coworkers demonstrated reduction of TGFβ levels in patients treated with N-acetylcysteine. This suggests that TGFβ levels are regulated by free oxygen radicals which were scavenged by N-acetylcysteine.

**Figure 1:**
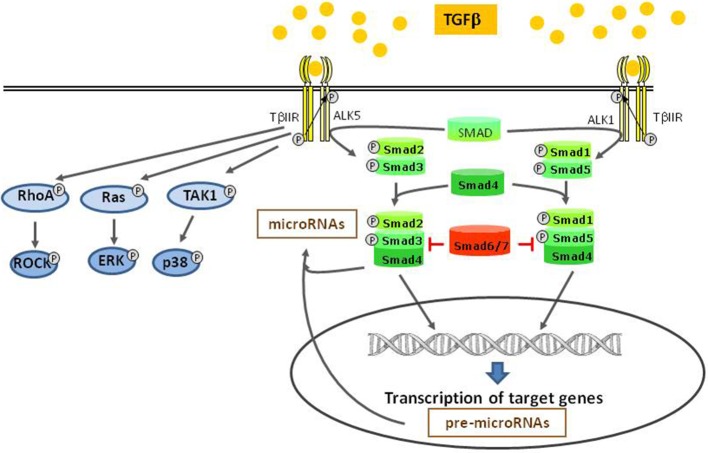
**TGFβ-signaling pathways**. After binding to TGFβ II receptor, different type I receptors (ALKs) can be activated and induce the canonical SMAD pathways. This pathway takes influence on miRNA modulation, either on the transcriptional level or by post transcriptional processing. Independent of the canonical pathway, other kinases are directly activated by the type II receptor.

Binding of TGFβ to its specific receptor type II (TβRII) enables phosphorylation and thus activation of type I receptors (TβIR), also known as activin receptor like kinase (ALK). The serin/threonine-kinase activity of this receptor enables phosphorylation and activation of transcription factors of the SMAD family (Heldin et al., 1997; Euler-Taimor and Heger, 2006). Different TβIR (ALK 1–7) can either activate the transcription factors SMAD2 and 3 or SMAD1 and 5. Thus, depending on the expression of these different TβIR types, a cell either responds with SMAD2/3 or SMAD1/5 activation (Wharton and Derynck, 2009). At first, dual activation of SMAD2/3 via ALK5 and SMAD1/5 via ALK1 by TGFβ was described in endothelial cells (Goumans et al., 2002), but it is now also found in other cell types (Figure 1). Together with the constitutively present SMAD4 these receptor-activated SMADs form heteromers that translocate to the nucleus and control SMAD-dependent gene transcription. This canonical SMAD-pathway can be abrogated by presence of inhibitory SMAD 6 or 7. Although the canonical SMAD-pathway is regarded as the main pathway of TGFβ-signaling, there exist also several non-canonical pathways. Mediated via the TGFβ-specific receptor II, kinases like TAK1, RhoA, p38, and ERK can be activated (Dobaczewski et al., 2011) (Figure 1). A relatively new and broad aspect of TGFβ-signaling now is added by its influence on microRNA expression. TGFβ-induced SMADs can bind to microRNA promoters thereby enhancing or reducing their transcription. In addition, SMADs can also contribute to post-translational microRNA processing by association with the Drosha-complex that is responsible for cutting pri-microRNAs into its active forms (Blahna and Hata, 2012).

Due to this broad spectrum of signaling points that can be targeted by TGFβ, an ample number of diverse functions of TGFβ are found. The cell type and the cellular environment determine the number and kind of TGFβ-receptors and the intracellular milieu, both of which have an impact on the cell reaction upon TGFβ stimulation. These complex signaling components of TGFβ may also be the reason for the different actions of TGFβ in ischemic-reperfused myocardium.

## Trapping TGFβ by soluble TGFβ receptors unmask protective and detrimental effects of TGFβ in myocardial infarction

As said above, several studies described upregulation of TGFβ and SMADs after myocardial infarction. These findings point to a role of TGFβ in the infarcted heart, and prompted studies using inhibitors of TGFβ/SMAD signaling in order to determine the role of this pathway in post-infarct remodeling.

For efficient blockade of TGFβ-signaling expression of a soluble TGFβ recetor (sTβIIR) was used. Ikeuchi et al. (2004) injected plasmids that encoded sTβIIR in the tibial muscle of mice, followed by electroporation to improve gene transfection. This single injection resulted in increased serum levels of sTβIIR reaching maximal expression levels 7–10 days after transfection. To determine the influence of this inhibitor on post-infarction remodeling, mice were transfected at different time points. These studies revealed different outcomes of TGFβ inhibition, depending on the time of inhibition. When mice were transfected 7 days before permanent coronary ligation, mortality rate up to 24 h of myocardial infarction was dramatically enhanced by TGFβ-inhibition, although infarct size did not change. Enhanced mortality in the sTβIIR-treated myocardial infarction group was accompanied by an exacerbated LV dysfunction and immune response, indicated by enhanced cytokine expression and infiltration of neutrophils. Interestingly, when transfection was performed at the same time as the ligation and 7 days thereafter, survival rate and infarct size 4 weeks after coronary ligation was not influenced by sTβIIR. Echocardiography revealed reduction of left ventricular dilatation and improved LV function due to sTβIIR. In addition, sTβIIR reduced myocyte cross sectional area and collagen volume fraction, thereby reducing myocardial hypertrophy in late remodeling. These findings indicate that enhancement of TGFβ-levels in ischemic hearts have protective characters in the early phase, but is detrimental in the late phase of post-myocardial infarction remodeling. Thus, timing of anti-TGFβ-treatment seems to be decisive for the outcome of an effective therapeutic use in myocardial infarction.

Another study that also used sTβIIR for TGFβ-inhibition in post-myocardial infarction comes from Okada et al. (2005). They used adenoviral transfection with the sTβIIR-gene. This produced a maximal increase in sTβIIR plasma levels 7 days after transfection of mice. In accordance with the results of Ikeuchi et al., Okada and coworkers found enhanced survival rates in sTβIIR-transfected mice 4 weeks after myocardial infarction when sTβIIR-transfection was performed 3 days after permanent coronary artery ligation. LV function was improved. Apoptosis was reduced among myofibroblasts, whereas sTβIIR did not change amounts of apoptotic endothelial cells or cardiomyocytes. The anti-apoptotic effect of sTβIIR may contribute to the enhanced amount of myofibroblasts in the post-myocardial infarction scar tissue, and these cells may improve geometry and thereby also function of the heart. Okada et al. also investigated effects of sTβIIR on the chronic phase in myocardial infarction by treating mice with sTβIIR 4 weeks after myocardial infarction. However, this treatment had no effect on survival, LV function or LV geometry.

Thus, in the same models of myocardial infarction, produced by permanent coronary artery ligation, application of sTβIIR has either protective or detrimental effects. The outcome of sTβIIR application mainly depends on the time point of sTβIIR treatment. Therefore, the investigations of Ikeuchie and Okada together suggest that there is only a narrow time window for an effective treatment of myocardial infarction with sTβIIR. It should not be used too early, as sTβIIR then abrogates protective TGFβ effects in the early post-infarction remodeling, and it should not be too late as it is no more effective when applied after scar formation has been completed.

## Detrimental effects of TGFβ: SMAD-signaling and apoptosis in myocardial infarction

The above mentioned investigations used sTβIIR to block all possible downstream signaling pathways of TGFβ. The broad action of this inhibitor may cause the pleiotropic effects in cardio-protection and dysfunction. For more target–oriented approaches inhibitors of specific TGFβ-signaling pathways can be used. In regard to this, first and foremost inhibitors of SMAD signaling have to be considered.

In isolated cardiomyocytes of adult rat, TGFβ has been shown to induce apoptotic cell death (Schneiders et al., 2005). This apoptosis induction could be blocked by prior transformation of cardiomyocytes with SMAD-decoy-oligonucleotides. These decoy-oligos scavenge SMADs intracellularly and thus interrupt SMAD-mediated TGFβ-effects. In addition, blocking TβIR by SB431542, a potent and specific inhibitor of ALK4, 5, and 7 (Inman et al., 2002), interrupted SMAD2 signaling and apoptosis induction due to TGFβ stimulation of cardiomyocytes (Heger et al., 2011). Apoptosis-related target genes of SMADs in cardiomyocytes have not been defined yet. However, in other cell types and tissues SMAD-regulated pro-apoptotic genes, like PUMA or Bim, have been defined (Spender et al., 2013 and Ha Thi et al., 2013), and may be possible target genes in cardiomyocytes. Due to the fact that TGFβ promotes apoptosis via SMAD-signaling in cardiomyocytes, this pathway may contribute to cardiomyocytes loss after myocardial infarction *in vivo*.

**Figure 2:**
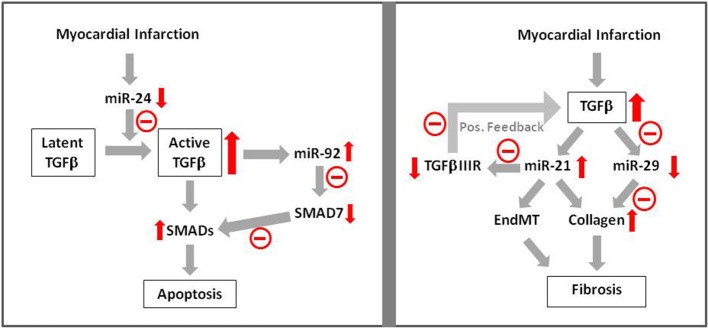
**MicroRNA signaling in TGFβ-induced apoptosis and fibrosis**. Apoptosis induction after myocardial infarction has been shown to be mediated via TGFβ —microRNA—SMAD signaling. Influence of microRNAs on fibrosis has been shown via enhancement of collagen synthesis and EndMT. Red arrows indicate enhancement or reduction of the respective signaling molecule. 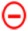 Indicates negative/reducing influence of this pathway.

Further evidence for this hypothesis comes from a recent study from Guo et al. (2014), demonstrating contribution of miR-24 to apoptosis after myocardial infarction. They observed downregulation of miR-24 and apoptosis induction after myocardial infarction. Cardiomyocyte-specific miR-24 overexpression in transgenic mice prevented myocardial infarction-induced apoptosis and improved cardiac function. In another study it has been shown that miR-24 prevents processing of latent TGFβ to its active form (Wang et al., 2012), indicating that reduction of TGFβ-levels may have contributed to reduced apoptosis after myocardial infarction. Besides miR-24 other microRNAs may also play a role in TGFβ-induced apoptosis after myocardial infarction. In H9c2 cells induction of apoptosis by hypoxia/reoxygenation was shown to be dependent on induction of miR92a (Zhang et al., 2014). Antagomirs against miR-92a abolished the apoptotic response and increased the levels of the inhibitory SMAD7, thereby indicating that suppression of SMAD-signaling may have contributed to inhibition of hypoxia/reoxygenation induced apoptosis. Thus, induction of microRNAs in ischemic-reperfused myocardium seems to boost TGFβ/SMAD-signaling in order to enhance apoptosis which then may contribute to adverse remodeling (Figure 2).

In several investigations preserved cardiac performance due to reduction of cardiomyocyte apoptosis during myocardial infarction has been shown. In those studies classical inhibitors of the apoptotic signaling cascades were used. Reduced infarct sizes and improved functions after myocardial infarction were found in Fas- or Bax-deficient mice (Hochhauser et al., 2003; Lee et al., 2003). Overexpression of anti-apoptotic proteins like Bcl2 or IAP (inhibitor of apoptosis) also reduced infarct size in hearts (Chen et al., 2001; Chua et al., 2007). Whether specific interference with the SMAD2/3 pathway indeed conveys protection against apoptosis in myocardial infarction *in vivo* and if this will improve survival rates in patients still has to be proven.

Just recently, a cardioprotective role in myocardial infarction has been shown for another TGFβ-family member, namely BMP2 (Ebelt et al., 2013). BMP2 mediates its signaling via TGFβ I-receptor types (ALK1, 2, or 3), resulting in activation of SMAD1/5/8. Now it has been proven that activation of this pathway by a single bolus injection of BMP2 post-myocardial infarction is able to reduce apoptotic cell death of cardiomyocytes and improves cardiac function, although mortality rates of mice were not affected.

**Figure 3:**
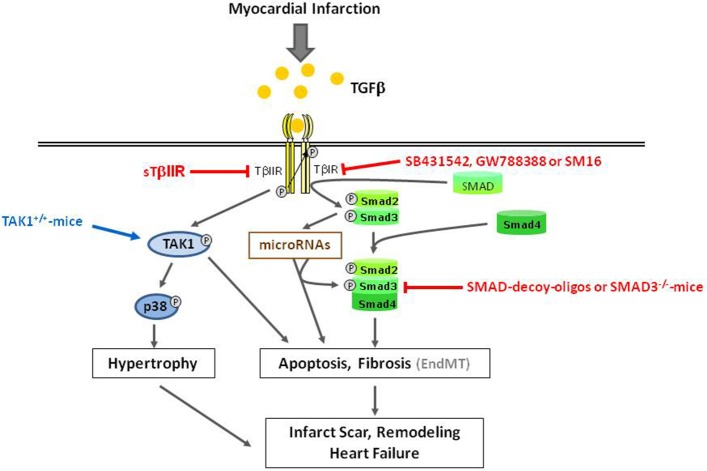
**Overview of detrimental TGFβ-induced effects in ischemic reperfused myocardium**. Gray arrows indicate physiological signaling pathways. Red and blue arrows/lines indicate pharmacological interventions used for characterization of signaling pathways.

Thus, with regards to apoptosis induction after myocardial infarction, SMAD family members may either be detrimental, as under TGFβ-stimulation (Figure 3), or confer protective effects as shown under BMP2-stimulation. The detrimental TGFβ-effects on cardiac apoptosis can be modulated by microRNAs (Figure 2).

## Detrimental effects of TGFβ: SMAD-signaling and fibrosis

A predominant role of TGFβ/SMAD signaling has been elucidated in the context of fibrosis induction after myocardial infarction. Enhancement of TGFβ levels after myocardial infarction always goes along with increases in fibrotic areas. Not only associations between these events have been described, but also direct involvement of TGFβ/SMADs has been shown. Using SMAD3-deficient mice, Bujak et al. (2007) have demonstrated reduced interstitial fibrosis post-myocardial infarction and prevention of diastolic dysfunction. Furthermore, in isolated fibroblasts of SMAD3-deficient mice TGFβ could no more enhance collagen synthesis. SMAD3-deficient fibroblasts exhibit reduced migratory potential, and reduced potential for transdifferentiation, that is consistent with a reduction in α-smooth muscle actin expressing myofibroblasts with reduced contractile function in SMAD3-deficient hearts after myocardial infarction (Dobaczewski et al., 2010). All these parameters indicate a strong impact of TGFβ/SMAD3 on fibrosis in post-myocardial infarction. Interestingly, SMAD3-deficiency did not alter the inflammatory response in the myocardium. Therefore, targeting the SMAD3 pathway may open a therapeutic window to reduce adverse fibrotic TGFβ-effects without affecting its positive actions in the early immune responses.

Another path for interference in the TGFβ/SMAD pathway has been applied in a recent study of Tan et al. (2010). They used the novel TβIR-inhibitor GW788388. When applied 1 week after coronary ligation in rats, SMAD2 activation, myofibroblast accumulation, collagen deposition, as well as systolic dysfunction was attenuated, while TGFβ-levels remained the same. No influence of GW7889388 on macrophage accumulation, that is essential to facilitate initial wound healing in the infarct area, was found. Similar anti-fibrotic effects of another orally applied TβIR-inhibitor, SM16, were recently demonstrated in a pressure-overload model after aortic banding (Engebretsen et al., 2014). However, SM16-treated mice exhibited increased mortality rates under pressure overload due to aortic rupture. Use of other TβIR-inhibitors resulted in inflammatory heart valve lesions (Anderton et al., 2011). Thus, the therapeutic potential of pharmacologic inhibition of TβIR to reduce cardiac fibrosis after myocardial infarction may be limited due to the mentioned adverse side effects (Figure 3).

Another opportunity to interfere with cardiac fibrosis due to TGFβ-induction is interference in microRNA pathways. van Rooij et al. (2008) have shown that TGFβ downregulates miR-29a in cardiac fibroblasts and this goes along with enhanced collagen expression. After myocardial infarction miR-29 expression is downregulated. This may contribute to enhanced myocardial fibrosis. Even stronger evidence for influence of microRNAs on TGFβ-induced fibrosis in myocardial infarction can be attributed to miR-21. It is upregulated after myocardial infarction and its addition to fibroblasts enhances collagen synthesis. Enhancement of miR-21 expression by TGFβ is shown. miR-21 itself represses TGFβRIII, a repressor of TGFβ-expression. Thus upon induction of TGFβ/miR-21 after myocardial infarction, TGFβIIIR expression is reduced. Lack of TGFβIIIR then facilitates further upregulation of TGFβ/miR-21 thereby boosting activation of fibroblasts and progression of cardiac fibrosis after myocardial infarction (Liang et al., 2012) (Figure 2).

Interestingly, cardiac fibrosis is not only established by fibroblast proliferation of resident cells, but also recruits fibroblasts from other cell sources, such as endothelial cells. After pressure overload about 30 % of fibroblasts are of endothelial origin (Zeisberg et al., 2007). These cells pass through endothelial mesenchymal transition (EndMT), so that they still express endothelial markers, such as CD31, but also gain fibroblast characteristics (Zeisberg et al., 2007). EndMT can be induced by TGFβ1 and is abrogated in SMAD3 deficient mice, or by BMP7 treatment (Zeisberg et al., 2003). TGFβ-induced EndMT may contribute to fibrosis after myocardial infarction, since under hypoxic conditions endothelial cells release bio-active TGFβ (Akman et al., 2001). In addition, miR-21, that is found to be induced after myocardial infarction (Liang et al., 2012), was identified as a mediator of TGFβ-induced EndMT (Kumarswamy et al., 2012). Furthermore, our own recent findings revealed TGFβ-dependent EndMT in microvascular endothelial cells under hypoxic conditions (unpublished data) providing even stronger evidence for the occurrence of EndMT after myocardial infarction.

These effects of TGFβ on fibrosis development in myocardial infarction contribute to enhanced myocardial stiffness and dysfunction.

## Detrimental effects of TGFβ: TAK1 and myocardial infarction

Besides the classical signaling pathway via SMADs, TGFβ can directly activate the TGFβ-receptor TGFβ-activated kinase (TAK1). This kinase influences events in ischemic-reperfused myocardium, and is related to hypertrophic responses.

The development of cardiac hypertrophy after myocardial infarction can also be supported by TGFβ. Although in isolated adult cardiomycoytes TGFβ does not directly stimulate hypertrophic growth, it can enhance the hypertrophic responsiveness of cardiomyocytes to β-adrenergic stimulation (Schlüter et al., 1995). Hypertrophy and enhanced TGFβ-levels most often correlate with TAK1 induction. This indicates that TAK1 may be involved in hypertrophy promoting effects of TGFβ. Support for this hypothesis comes from findings of Zhang et al. (2000), who demonstrate that an activating mutation of TAK1 expressed in myocardium of transgenic mice was sufficient to produce cardiac hypertrophy, but it also induces fibrosis and cardiac dysfunction. Activation of the TGFβ_1_-TAK1-p38 MAPK pathway is also found after myocardial infarction and parallels the transcriptional upregulation of cardiac markers for ventricular hypertrophy, beta-myosin heavy chain and atrial natriuretic peptide, thereby indicating that this pathway may be involved in hypertrophic growth processes after myocardial infarction (Matsumoto-Ida et al., 2006) (Figure 3). However, direct evidence for TAK1-mediated hypertrophic processes in myocardial infarction has not yet been demonstrated.

Just recently it was shown that disruption of the TAB1 (transforming growth factor-β (TGFβ-activated protein kinase 1 (TAK1)-binding protein 1) /p38α interaction by cell-permeable peptides limits myocardial ischemia/reperfusion injury by reduction of apoptotic cardiomyocytes and infarct size when applied 30 min before reperfusion (Wang et al., 2013). Thus, TAK1 might be involved not only in hypertrophy, but also fibrosis and apoptosis after myocardial infarction. In this context, the specific contributions of p38 kinases seem to mediate the non-canonical effects of TGFβ/TAK-signaling. The pathophysiological role of p38 is summarized in detail in a review by Marber et al. (2011), and, therefore, will not be further discussed at this point.

## Protective TGFβ-effects

Until now, we discussed the numerous investigations that demonstrated detrimental effects of TGFβ in ischemic reperfused myocardium. However, there are also studies that showed cardioprotective effects of TGFβ1. As mentioned above, Ikeuchi et al. (2004) revealed a protective time window for TGFβ in the early phase of myocardial infarction that was related to reduction of inflammatory responses in presence of TGFβ. Whereas in the later phase transient blockade of TGFβ was protective, indicating a detrimental role of TGFβ in late myocardial infarction. The exogenous application of TGFβ prior to reperfusion protected against cardiac injury, presumably by inhibiting neutrophils from adhering to endothelium (Lefer et al., 1993). Frantz et al. (2008) described a protective role of TGFβ also at later time points after myocardial infarction. Permanent blockade of TGFβ increased mortality rates and worsened left ventricular remodeling. This protection went along with alterations in the myocardial matrix. Thus, not only timing but also duration of TGFβ inhibition seems to influence the impact of TGFβ in ischemic reperfused myocardium.

To elicit the signaling mechanisms of protective TGFβ actions several studies on isolated cells or hearts were performed. In isolated cardiomyocytes TGFβ was shown to prevent hypoxia/reperfusion induced cell death, either attributed to apoptosis or necrosis (Baxter et al., 2001; Dandapat et al., 2008). Baxter and coworkers applied TGFβ at the beginning of reoxygenation, thereby indicating protection against reperfusion injury. This TGFβ-induced protection was conveyed by activation of ERK that belongs to the reperfusion injury salvage kinase (RISK)-pathway (Hausenloy and Yellon, 2004).

Those findings about protective roles of TGFβ against cardiac cell death are in contrast to findings of apoptosis induction by TGFβ in cardiomyocytes. There are two main differences between these studies. Protective effects of TGFβ are found in neonatal or HL1 cells, whereas apoptosis promoting effects are described in adult cardiomyocytes. Thus, the age of animals or cardiomyocytes may change the TGFβ-responsiveness. Furthermore, the hypoxic, oxygen radical enriched environment might influence the response, as it is shown in HL1 cells. There, TGFβ acts via an anti-oxidative mechanism under hypoxic conditions (Dandapat et al., 2008).

Another interesting field of TGFβ-induced cardioprotection comes from stem cell research. Here induction of the regenerative myogenic differentiation potential of bone marrow derived stem cells has been demonstrated (Li et al., 2005). Stem cells that were pretreated with TGFβ and then implanted intramyocardially had an enhanced regeneration potential in infarcted myocardium and contributed to functional improvements after myocardial infarction.

Only few studies had a deeper look at the signaling molecules that convey TGFβ-induced protection against myocardial injury. From these studies it can be said that non-canonical TGFβ-pathways seem to play a predominant role, mediated via ERK, since in intact rat hearts the protective role of TGFβ-infusion in early reperfusion was blocked by an ERK inhibitor (Baxter et al., 2001).

## Conclusion remarks

In conclusion, while TGFβ, applied or released at early times in myocardial infarction, act cardioprotective, most presumably via the non-canonical pathway; main influences of TGFβ, released at later time points after myocardial infarction, are the induction of apoptosis, hypertrophy, and fibrosis. These processes are conveyed via the SMAD2/3 signaling pathway, microRNAs and TAK1. Thus, targeting the classical SMAD or TAK pathways, or influencing SMAD/microRNA actions may provoke the best options for protection against TGFβ-induced adverse ischemic remodeling processes resulting in improved heart function after myocardial infarction.

### Conflict of interest statement

The author declares that the research was conducted in the absence of any commercial or financial relationships that could be construed as a potential conflict of interest.

## References

[B1] Akman H. O. Zhang H. Siddiqui M. A. Solomon W. Smith E. L. Batuman O. A. (2001). Response to hypoxia involves transforming growth factor-beta2 and Smad proteins in human endothelial cells. Blood 98, 3324–3331 10.1182/blood.V98.12.3324 11719370

[B2] Anderton M. J. Mellor H. R. Bell A. Sadler C. Pass M. Powell S.. (2011). Induction of heart valve lesions by small-molecule ALK5 inhibitors. Toxicol. Pathol. 39, 916–924. 10.1177/0192623311416259 21859884

[B3] Baxter G. F. Mocanu M. M. Brar B. K. Latchman D. S. Yellon D. M. (2001). Cardioprotective effects of transforming growth factor-beta1 during early reoxygenation or reperfusion are mediated by p42/p44 MAPK. J. Cardiovasc. Pharmacol. 38, 930–939. 10.1097/00005344-200112000-00015 11707697

[B4] Blahna M. T. Hata A. (2012). Smad-mediated regulation of microRNA biosynthesis. FEBS Lett. 586, 1906–1912. 10.1016/j.febslet.2012.01.041 22306316 PMC4429768

[B5] Bujak M. Ren G. Kweon H. J. Dobaczewski M. Reddy A. Taffet G.. (2007). Essential role of Smad3 in infarct healing and in the pathogenesis of cardiac remodeling. Circulation, 116(19), 2127-2138. 10.1161/CIRCULATIONAHA.107.704197 17967775

[B6] Chen Z. Chua C. C. Ho Y. S. Hamdy R. C. Chua B. H. (2001). Overexpression of Bcl-2 attenuates apoptosis and protects against myocardial I/R injury in transgenic mice. Am. J. Physiol. Heart Circ. Physiol. 280, H2313–H2320. 11299236 10.1152/ajpheart.2001.280.5.H2313

[B7] Chua C. C. Gao J. Ho Y. S. Xiong Y. Xu X. Chen Z.. (2007). Overexpression of IAP-2 attenuates apoptosis and protects against myocardial ischemia/reperfusion injury in transgenic mice. Biochim. Biophys. Acta 1773, 577–583. 10.1016/j.bbamcr.2007.01.007 17321613 PMC2709410

[B8] Dandapat A. Hu C. P. Li D. Liu Y. Chen H. Hermonat P. L.. (2008). Overexpression of TGFbeta1 by adeno-associated virus type-2 vector protects myocardium from ischemia-reperfusion injury. Gene Ther. 15, 415–423. 10.1038/sj.gt.3303071 18004403

[B9] Dobaczewski M. Bujak M. Li N. Gonzalez-Quesada C. Mendoza L. H. Wang X. F. (2010). Smad3 signaling critically regulates fibroblast phenotype and function in healing myocardial infarction. Circ. Res. 107, 418–428 10.1161/CIRCRESAHA.109.216101 20522804 PMC2917472

[B10] Dobaczewski M. Chen W. Frangogiannis N. G. (2011). Transforming growth factor (TGF)-β signaling in cardiac remodeling. J. Mol. Cell. Cardiol. 51, 600–606. 10.1016/j.yjmcc.2010.10.033 21059352 PMC3072437

[B11] Ebelt H. Hillebrand I. Arlt S. Zhang Y. Kostin S. Neuhaus H.. (2013). Treatment with bone morphogenetic protein 2 limits infarct size after myocardial infarction in mice. Shock 39, 353–360. 10.1097/SHK.0b013e318289728a 23376954

[B12] Edgley A. J. Krum H. Kelly D. J. (2012). Targeting fibrosis for the treatment of heart failure: a role for transforming growth factor-β. Cardiovasc. Ther. 30, e30–e40. 10.1111/j.1755-5922.2010.00228.x 21883991

[B13] Engebretsen K. V. Skårdal K. Bjørnstad S. Marstein H. S. Skrbic B. Sjaastad I.. (2014). Attenuated development of cardiac fibrosis in left ventricular pressure overload by SM16, an orally active inhibitor of ALK5. J. Mol. Cell. Cardiol. 76C, 148–157. 10.1016/j.yjmcc.2014.08.008 25169971

[B14] Euler-Taimor G. Heger J. (2006). The complex pattern of SMAD signaling in the cardiovascular system. Cardiovasc. Res. 69, 15–25. 10.1016/j.cardiores.2005.07.007 16107248

[B15] Frantz S. Hu K. Adamek A. Wolf J. Sallam A. Maier S. K.. (2008). Transforming growth factor beta inhibition increases mortality and left ventricular dilatation after myocardial infarction. Basic. Res. Cardiol. 103, 485–492. 10.1007/s00395-008-0739-7 18651091

[B16] Goumans M. J. Valdimarsdottir G. Itoh S. Rosendahl A. Sideras P. ten Dijke P. (2002). Balancing the activation state of the endothelium via two distinct TGF-beta type I receptors. EMBO J. 21, 1743–1753. 10.1093/emboj/21.7.1743 11927558 PMC125949

[B17] Guo C. Deng Y. Liu J. Qian L. (2014). Cardiomyocyte-specific role of miR-24 in promoting cell survival. J. Cell. Mol. Med. 19, 103–112. 10.1111/jcmm.12393 25352422 PMC4288354

[B18] Hao J. Ju H. Zhao S. Junaid A. Scammell-La Fleur T. Dixon I. M. (1999). Elevation of expression of Smads 2, 3, and 4, decorin and TGF-beta in the chronic phase of myocardial infarct scar healing. J. Mol. Cell. Cardiol. 31, 667–678. 10.1006/jmcc.1998.0902 10198196

[B19] Ha Thi H. T. Lim H. S. Kim J. Kim Y. M. Kim H. Y. Hong S. (2013). Transcriptional and post-translational regulation of Bim is essential for TGF-β and TNF-α-induced apoptosis of gastric cancer cell. Biochim. Biophys. Acta 1830, 3584–3592. 10.1016/j.bbagen.2013.03.006 23500081

[B20] Hausenloy D. J. Yellon D. M. (2004). New directions for protecting the heart against ischaemia–reperfusion injury: targeting the Reperfusion Injury Salvage Kinase (RISK)-pathway. Cardiovasc. Res. 61, 448–460. 10.1016/j.cardiores.2003.09.024 14962476

[B21] Heger J. Warga B. Abdallah Y. Meyering B. Schlüter K. D. Euler G. (2011). TGFβ receptor activation enhances cardiac apoptosis via SMAD activation and concomitant NO release. J. Cell. Physiol. 226, 2683–2690. 10.1002/jcp.22619 21792926

[B22] Heldin C. H. Miyazono K. ten Dijke P. (1997). TGF-beta signalling from cell membrane to nucleus through SMAD proteins. Nature 390, 465–471. 10.1038/37284 9393997

[B23] Hochhauser E. Kivity S. Offen D. Maulik N. Otani H. Barhum Y.. (2003). Bax ablation protects against myocardial ischemia-reperfusion injury in transgenic mice. Am. J. Physiol. Heart Circ. Physiol., 284, H2351–H2359. 10.1152/ajpheart.00783.2002 12742833

[B24] Huntgeburth M. Tiemann K. Shahverdyan R. Schlüter K. D. Schreckenberg R. Gross M. L. (2011). Transforming growth factor β1 oppositely regulates the hypertrophic and contractile response to β-adrenergic stimulation in the heart. PLoS ONE 6:e26628 10.1371/journal.pone.0026628 22125598 PMC3219639

[B25] Ikeuchi M. Tsutsui H. Shiomi T. Matsusaka H. Matsushima S. Wen J.. (2004). Inhibition of TGF-beta signaling exacerbates early cardiac dysfunction but prevents late remodeling after infarction. Cardiovasc. Res. 64, 526–535. 10.1016/j.cardiores.2004.07.017 15537506

[B26] Inman G. J. Nicolás F. J. Callahan J. F. Harling J. D. Gaster L. M. Reith A. D.. (2002). SB-431542 is a potent and specific inhibitor of transforming growth factor-beta superfamily type I activin receptor-like kinase (ALK) receptors ALK4, ALK5, and ALK7. Mol. Pharmacol. 62, 65–74. 10.1124/mol.62.1.65 12065756

[B27] Kumarswamy R. Volkmann I. Jazbutyte V. Dangwal S. Park D. H. Thum T. (2012). Transforming growth factor-β-induced endothelial-to-mesenchymal transition is partly mediated by microRNA-21. Arterioscler. Thromb. Vasc. Biol. 32, 361–369. 10.1161/ATVBAHA.111.234286 22095988

[B28] Lee P. Sata M. Lefer D. J. Factor S. M. Walsh K. Kitsis R. N. (2003). Fas pathway is a critical mediator of cardiac myocyte death and MI during ischemia-reperfusion *in vivo*. Am. J. Physiol. Heart Circ. Physiol. 284, H456–H463. 10.1152/ajpheart.00777.2002 12414449

[B29] Lefer A. M. Ma X. L. Weyrich A. S. Scalia R. (1993). Mechanism of the cardioprotective effect of transforming growth factor beta 1 in feline myocardial ischemia and reperfusion. Proc. Natl. Acad. Sci. U.S.A. 90, 1018–1022. 10.1073/pnas.90.3.1018 8381531 PMC45802

[B30] Li Q. Xu Y. Li X. Guo Y. Liu G. (2012). Inhibition of Rho-kinase ameliorates myocardial remodeling and fibrosis in pressure overload and myocardial infarction: role of TGF-β 1-TAK1. Toxicol. Lett. 211, 91–97. 10.1016/j.toxlet.2012.03.006 22465603

[B31] Li T. S. Hayashi M. Ito H. Furutani A. Murata T. Matsuzaki M. (2005). Regeneration of infarcted myocardium by intramyocardial implantation of *ex vivo* transforming growth factor-beta-preprogrammed bone marrow stem cells. Circulation 111, 2438–2445 10.1161/01.CIR.0000167553.49133.81 15883211

[B32] Liang H. Zhang C. Ban T. Liu Y. Mei L. Piao X.. (2012). A novel reciprocal loop between microRNA-21 and TGFβRIII is involved in cardiac fibrosis. Int. J. Biochem. Cell. Biol. 44, 2152–2160. 10.1016/j.biocel.2012.08.019 22960625

[B33] Marber M. S. Rose B. Wang Y. (2011). The p38 mitogen-activated protein kinase pathway–a potential target for intervention in infarction, hypertrophy, and heart failure. J. Mol. Cell. Cardiol. 51, 485–490. 10.1016/j.yjmcc.2010.10.021 21062627 PMC3061241

[B34] Matsumoto-Ida M. Takimoto Y. Aoyama T. Akao M. Takeda T. Kita T. (2006). Activation of TGF-beta1-TAK1-p38 MAPK pathway in spared cardiomyocytes is involved in left ventricular remodeling after myocardial infarction in rats. Am. J. Physiol. Heart Circ. Physiol. 290, H709–H715. 10.1152/ajpheart.00186.2005 16183734

[B35] Okada H. Takemura G. Kosai K. Li Y. Takahashi T. Esaki M.. (2005). Postinfarction gene therapy against transforming growth factor-beta signal modulates infarct tissue dynamics and attenuates left ventricular remodeling and heart failure. Circulation 111, 2430–2437. 10.1161/01.CIR.0000165066.71481.8E 15867170

[B36] Schlüter K. D. Zhou X. J. Piper H. M. (1995). Induction of hypertrophic responsiveness to isoproterenol by TGF-beta in adult rat cardiomyocytes. Am. J. Physiol. 269(Pt 1), C1311–C1316. 7491923 10.1152/ajpcell.1995.269.5.C1311

[B37] Schneiders D. Heger J. Best P. Piper H. M. Taimor G. (2005). SMAD proteins are involved in apoptosis induction in ventricular cardiomyocytes. Cardiovasc. Res. 67, 87–96. 10.1016/j.cardiores.2005.02.021 15949472

[B38] Spender L. C. Carter M. J. O'Brien D. I. Clark L. J. Yu J. Michalak E. M. (2013). Transforming growth factor-β directly induces p53-up-regulated modulator of apoptosis (PUMA) during the rapid induction of apoptosis in myc-driven B-cell lymphomas. J. Biol. Chem. 288, 5198–5209 10.1074/jbc.M112.410274 23243310 PMC3576124

[B39] Talasaz A. H. Khalili H. Jenab Y. Salarifar M. Broumand M. A. Darabi F. (2013). N-Acetylcysteine effects on transforming growth factor-β and tumor necrosis factor-α serum levels as pro-fibrotic and inflammatory biomarkers in patients following ST-segment elevation myocardial infarction. Drugs R D 13, 199–205 10.1007/s40268-013-0025-5 24048773 PMC3784054

[B40] Tan S. M. Zhang Y. Connelly K. A. Gilbert R. E. Kelly D. J. (2010). Targeted inhibition of activin receptor-like kinase 5 signaling attenuates cardiac dysfunction following myocardial infarction. Am. J. Physiol. Heart Circ. Physiol. 298, H1415–H1425 10.1152/ajpheart.01048.2009 20154262

[B41] van Rooij E. Sutherland L. B. Thatcher J. E. DiMaio J. M. Naseem R. H. Marshall W. S.. (2008). Dysregulation of microRNAs after myocardial infarction reveals a role of miR-29 in cardiac fibrosis. Proc. Natl. Acad. Sci. U.S.A. 105, 13027–13032. 10.1073/pnas.0805038105 18723672 PMC2529064

[B42] Vilahur G. Juan-Babot O. Peña E. Oñate B. Casaní L. Badimon L. (2011). Molecular and cellular mechanisms involved in cardiac remodeling after acute myocardial infarction. J. Mol. Cell. Cardiol. 50, 522–533 10.1016/j.yjmcc.2010.12.021 21219908

[B43] Wang J. Huang W. Xu R. Nie Y. Cao X. Meng J.. (2012). MicroRNA-24 regulates cardiac fibrosis after myocardial infarction. J. Cell. Mol. Med. 16, 2150–2160. 10.1111/j.1582-4934.2012.01523.x 22260784 PMC3822985

[B44] Wang Q. Feng J. Wang J. Zhang X. Zhang D. Zhu T.. (2013). Disruption of TAB1/p38α interaction using a cell-permeable peptide limits myocardial ischemia/reperfusion injury. Mol. Ther. 21, 1668–1677. 10.1038/mt.2013.90 23877036 PMC3776642

[B45] Wharton K. Derynck R. (2009). TGFbeta family signaling: novel insights in development and disease. Development 136, 3691–3697. 10.1242/dev.040584 19855012

[B46] Zeisberg E. M. Tarnavski O. Zeisberg M. Dorfman A. L. McMullen J. R. Gustafsson E.. (2007). Endothelial-to-mesenchymal transition contributes to cardiac fibrosis. Nat. Med. 13, 952–961. 10.1038/nm1613 17660828

[B47] Zeisberg M. Hanai J. Sugimoto H. Mammoto T. Charytan D. Strutz F.. (2003). BMP-7 counteracts TGF-beta1-induced epithelial-to-mesenchymal transition and reverses chronic renal injury. Nat. Med. 9, 964–968. 10.1038/nm888 12808448

[B48] Zhang B. Zhou M. Li C. Zhou J. Li H. Zhu D.. (2014). MicroRNA-92a inhibition attenuates hypoxia/reoxygenation-induced myocardiocyte apoptosis by targeting Smad7. PLoS ONE 9:e100298. 10.1371/journal.pone.0100298 24941323 PMC4062536

[B49] Zhang D. Gaussin V. Taffet G. E. Belaguli N. S. Yamada M. Schwartz R. J. (2000). TAK1 is activated in the myocardium after pressure overload and is sufficient to provoke heart failure in transgenic mice. Nat. Med. 6, 556–563 10.1038/75037 10802712

